# Enterocutaneous Fistula: Open Repair after Unsuccessful Stenting—A Case Report

**DOI:** 10.3390/medicina58020223

**Published:** 2022-02-02

**Authors:** Valerija Mosenko, Saulius Jurevičius, Audrius Šileikis

**Affiliations:** Medical Faculty, Vilnius University, LT-03101 Vilnius, Lithuania; saulius.jurevicius@rvul.lt (S.J.); audrius.sileikis@santa.lt (A.Š.)

**Keywords:** enterocutaneous fistula, self-expanding metal stents, gastrointestinal surgery

## Abstract

Enterocutaneous fistula (ECF) is an abnormal connection between the gastrointestinal tract and the skin; by some estimates, it represents 88.2% of all fistulae. It can either develop spontaneously due to underlying malignancy, inflammatory bowel disease, radiation exposure, or, more commonly, as a complication of gastrointestinal surgery. A 75-year-old woman was treated for a small bowel enterocutaneous fistula that developed after laparoscopic cholecystectomy using a HANAROSTENT self-expanding metal stent (SEMS) to cover the fistula. Seven months later, the patient was discharged. For the following 2 years, the patient refused the reconstructive surgery until stent obstruction occurred. After optimizing the patient’s nutritional status, laparotomy and small bowel resection were performed successfully. The use of SEMS in fistulas of the lower gastrointestinal tract is a heavily debated and fairly under-researched topic, especially in the context of enterocutaneous fistulas. No international guidelines officially recommend using SEMS in the small bowel ECF.

## 1. Introduction

Enterocutaneous fistula (ECF) is an abnormal connection between the gastrointestinal tract and the skin [1]; by some estimates, it represents 88.2% of all fistulae [2]. It can either develop spontaneously due to underlying malignancy, inflammatory bowel disease, radiation exposure, or, more commonly, as a complication of gastrointestinal surgery [3]. Small enteric fistulas (<1 cm) as well as long fistulas (>2 cm) are more likely to close spontaneously [4,5]. The main danger of ECFs are wound infection and sepsis as well as considerable loss of intestinal fluids, which may cause malnutrition, dehydration, and electrolyte imbalance [6]. The use of SEMS in fistulas of the lower gastrointestinal tract is a heavily debated and fairly under-researched topic. In this article, we present the case of using SEMS in ECF as a “bridge” to definitive surgery.

## 2. Case Report

**Figure 1:**
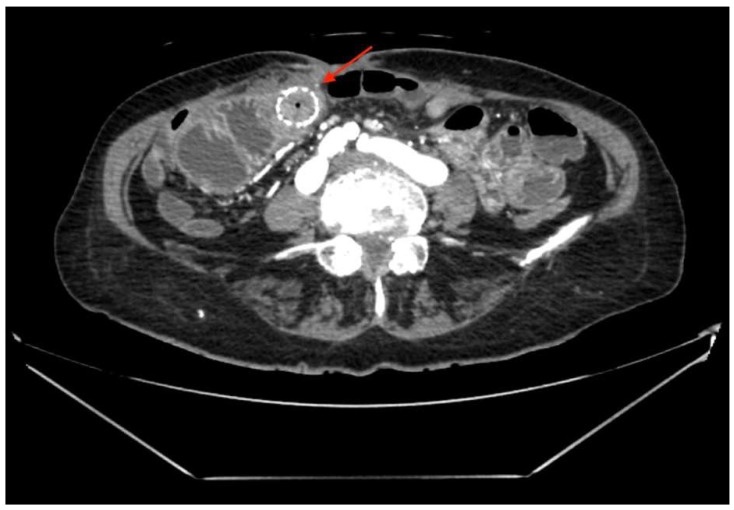
A small bowel stent with fibrous/tumor-like changes around it (red arrow).

A 75-year-old woman presented to the ER with right upper quadrant pain, nausea, and vomiting due to acute cholecystitis. The history was significant for an open right hemicolectomy that was performed 6 years before this admission due to colon adenocarcinoma. Threee days after the laparoscopic cholecystectomy, the inflammation markers were found to be elevated (WBC 11.36 × 10^9^/L, CRP 213 mg/L) and a diffuse peritonitis was identified. That same day, an exploratory laparotomy was performed. During the surgery, the small bowel was found to be perforated from suspected thermal injury (about 80 cm from plica duodenojejunalis), which was reconstructed successfully by primary bowel suture. Seven days after the surgery, a small intestinal eventration with a renewed bowel perforation was identified and a conservative treatment was initiated. Despite conservative treatment, the small intestinal fistula increased and eventually its ends separated, resulting in two open intestinal lobes. More than a month after the total parenteral feeding was initiated, the HANAROSTENT TLD-20110-230 (a single-use duodenal pyloric partially covered self-expanding metal stent) was inserted at both ends of the small intestine and fixed with single sutures to restore intestinal integrity. Two weeks after the implantation, the bowel fistula reoccurred; however, the output was noted to be comparably lower. The metallic stent was left in place for a longer time despite manufacturer’s recommendations, to act as a metal carcass to facilitate the healing of the external fistula. Seven months later, the patient was discharged after the total closure of the fistula (verified by upper GI and small bowel series), while being able to tolerate a regular feeding regimen. The follow-up appointment was scheduled to remove the stent, but unfortunately, the patient did not show up for the appointment. For the following 2 years, the patient was being intermittently hospitalized to a different hospital, due to mechanical bowel obstructions, which were treated conservatively due to the patient refusing surgery. Two years after the initial discharge from the primary hospital, the patient was admitted into our care due to acute bowel obstruction. The metabolic panel showed signs of chronic malnutrition with significant hypoproteinemia (29.7 g/L) and hypoalbuminemia (56.7 g/L), with a BMI of 20.76 kg/m^2^. Abdominal CT (Figure 1) showed a small bowel stent with fibrous/tumor-like changes around it (signs of small bowel obstruction).

**Figure 2:**
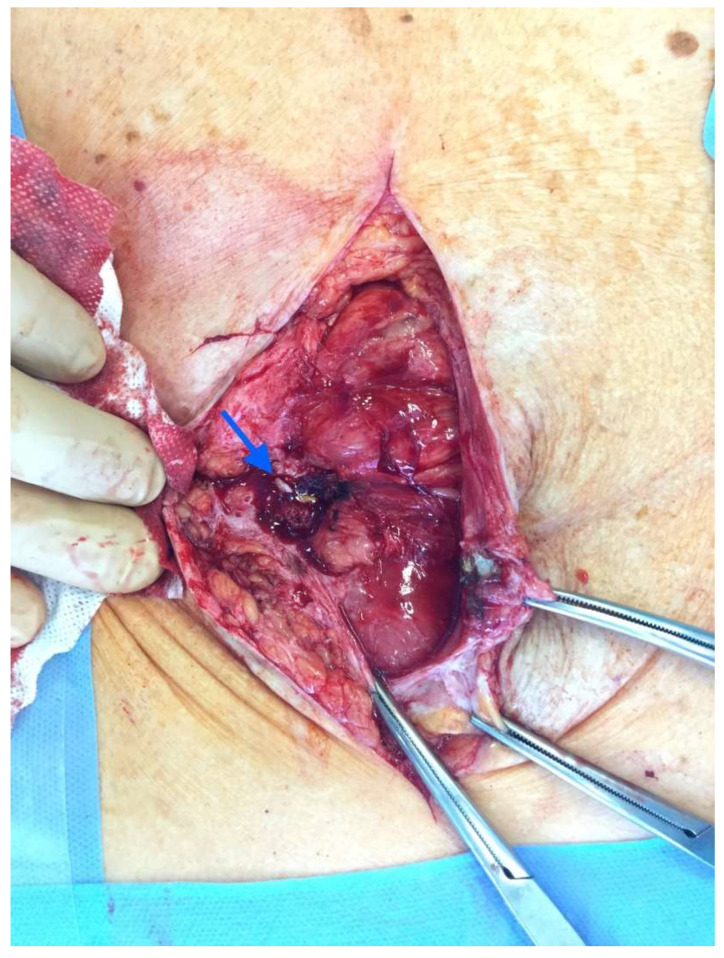
Enterocutaneous fistula in situ (blue arrow).

**Figure 3:**
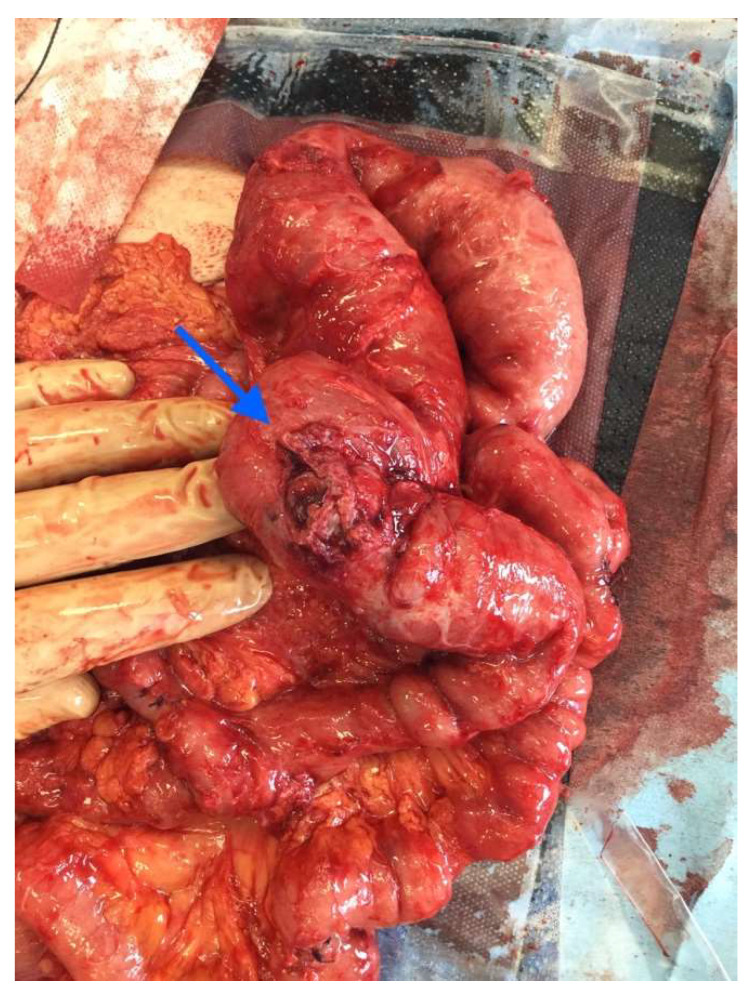
Enterocutaneous fistula seen in the mobilized small bowel (blue arrow).

**Figure 4:**
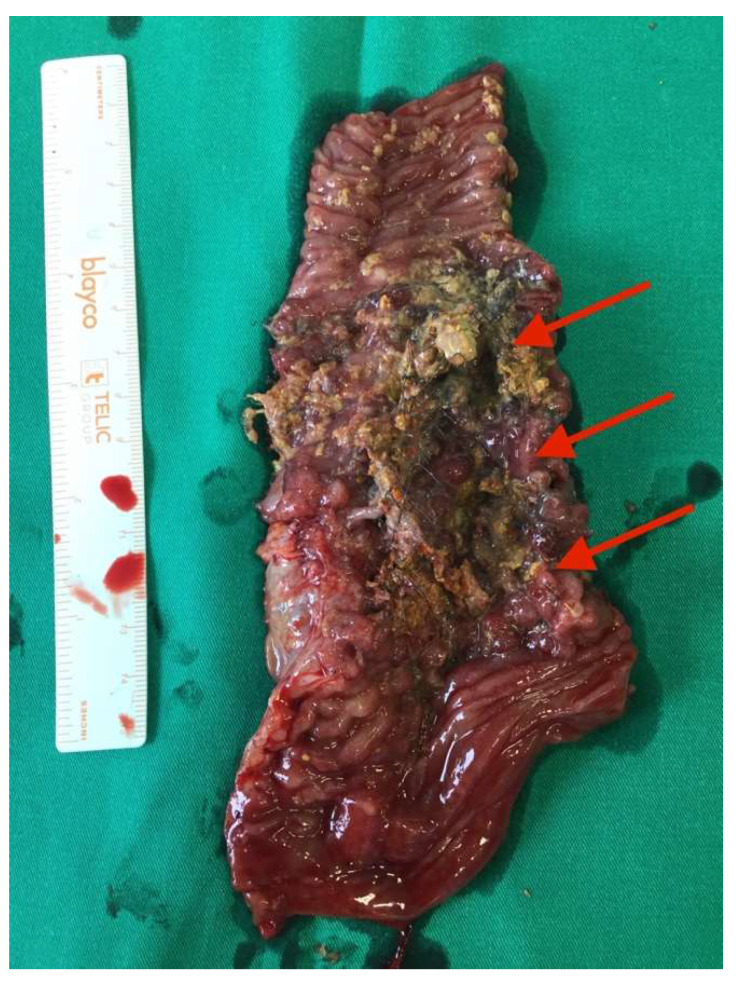
Metal self-expanding stent in the resected portion of the bowel (red arrows).

After the optimization of the patient’s nutritional status, a laparotomy, adhaeolysis, and jejunal resection were performed. A conglomerate consisting of the small intestine 80 cm from pl. duodenojejunalis was identified and a metal stent was palpated. The intestines 5 cm proximal to the blockage were found to be hypertrophic and considerably inflated (Figure 2 and Figure 3). The resection of 10 cm of the small bowel with the metal stent was performed and a jejunojejunal anastomosis was formed using a running 3-0 PDS suture (Figure 4).

Histopathological examination of the specimen (13.5 cm portion of the small bowel with an ingrown 7.5 cm metal stent and resection margins of 2 cm and 3.5 cm) revealed a diffuse submucosal lymphoplasmacytic infiltration with lymphoid follicles and polymorphonuclear leukocytes as well as ulcerated granulocytes.

After the surgery, the patient was monitored in the ICU for 2 days. The treatment consisted of adequate hydration and analgesia, vitamin therapy, thromboembolic prophylaxis, parenteral, and enteral nutrition. Due to persisting elevation of inflammation markers, the antibiotic therapy of Sol. Tazobactam/Piperacilin 4.5 × q8 was initiated.

### Follow-Up

The patient was discharged after 32 days in good health. At a 3-month follow-up, the patient presented with no complaints, had gained additional weight (6 kg, BMI 24.43 kg/m^2^), and showed no signs of hypokalemia or hypoproteinemia.

## 3. Discussion

In this case, the etiology of ECF was diathermy injury after cholecystectomy and adhesiolysis. Unfortunately, the exact incidence of enteric injury during cholecystectomy is unknown [7], and studies reporting on this topic are rare. Gupta et al. [8] observed enteric injury in 2 (4.76%) patients. Given the proximity of the duodenum and colon to the gall bladder, they are prone to injury during adhesiolysis or due to inadvertent diathermy trauma [9]. The detection of those injuries can be delayed, as they are rare and usually unexpected. 

The management of ECF requires a multidisciplinary and often rather aggressive approach to minimize the morbidity and mortality of the condition. The main therapeutic goals include sepsis control, attentive wound care, and the optimization of the patient’s nutritional status [6,10]. For sepsis control, the improved outcomes are achieved through volume resuscitation, nutritional support, and electrolyte imbalance correction as well as intravenous antibiotics and drainage of intra-abdominal abscesses. An important aspect of care is utilizing both parenteral and enteral nutritional support as well as aggressive wound management and timely surgical intervention. Surgery is usually recommended at least 3 months after the injury. Despite timely implementation of the aforementioned management steps, the mortality rates range from 5 to 29% overall, with surgical mortality up to 3.5% [4,6,11,12]. In this case, the placement of the stent was a difficult choice made out of necessity due to the poor overall condition of the patient. The enteral injury was detected only on the 3rd day post-laparoscopic cholecystectomy, when the abdominal wall phlegmon was already present and the enteral contents were escaping through the infraumbilical incision. The complete adhaeolysis was not performed initially, which unfortunately led to a leaking enteral suture. Once the relaparotomy was performed it became apparent that the fistula was situated just behind the aponeurosis and subsequently migrated into the hypodermal layer. After the surgery, a total parenteral nutrition was initiated and continued for 6 weeks, before the decision to implant SEMS to cover the fistula was made, as there were no signs of spontaneous closure present and the patient’s condition remained critical. Although the primary team considered other management strategies, none of them were suitable for this patient. T-tube drainage and stoma formation were excluded due to them leaving the “high” fistula open, which has been proven to be detrimental to patients because of the high-volume output of the intestinal contents and subsequent malnourishment. The resection of the fistula and anastomosis may have been beneficial; however, the primary surgical team as well as the patient were hoping to avoid the resection of the intestine for as long as possible. The long term TPN was administered for 7 months. Due to the concern of the foreign body in the intestinal tract, it was recommended that the patient come back for follow up; however, the patient did not adhere to the recommendations and was intermittently treated conservatively for bowel obstruction every 6 months.

The placement of the stent in this patient provided as a temporary relief, providing an opportunity to optimize the patient’s nutritional status and prepare them for surgery, which was unfortunately postponed due to the patient’s concerns and refusal to adhere to recommendations. 

The use of SEMS in fistulas of the lower gastrointestinal tract is a heavily debated and fairly under-researched topic, especially in the context of enterocutaneous fistulas. No international guidelines officially recommend using SEMS in the small bowel ECF; therefore, no comparison of outcomes can be achieved.

## 4. Conclusions

Enterocutaneous fistula remains a dreaded surgical complication with high mortality and morbidity rates, despite the considerable advances in ECF management. The use of SEMS in fistulas of the lower gastrointestinal tract is a heavily debated and fairly under-researched topic, especially in the context of enterocutaneous fistulas. The controversial decisions made in the management of the patient in the described case report highlight the benefits and limitations of using it as a “bridging” therapy towards a definitive fistula treatment. More data needs to be published to evaluate the efficacy of this treatment strategy in small bowel fistulas.
